# Retrolaminar Block Versus Paravertebral Block for Pain Relief After Less-Invasive Lung Surgery: A Randomized, Non-Inferiority Controlled Trial

**DOI:** 10.7759/cureus.13597

**Published:** 2021-02-27

**Authors:** Takuji Sugiyama, Yuki Kataoka, Kazuo Shindo, Miki Hino, Kazumi Itoi, Yukihito Sato, Shiro Tanaka

**Affiliations:** 1 Department of Anesthesia, Hyogo Prefectural Amagasaki General Medical Center, Amagasaki, JPN; 2 Hospital Care Research Unit, Hyogo Prefectural Amagasaki General Medical Center, Amagasaki, JPN; 3 Department of Respiratory Surgery, Hyogo Prefectural Amagasaki General Medical Center, Amagasaki, JPN; 4 Department of Cardiology, Hyogo Prefectural Amagasaki General Medical Center, Amagasaki, JPN; 5 Department of Clinical Biostatistics, Graduate School of Medicine, Kyoto University, Kyoto, JPN

**Keywords:** paravertebral block, retrolaminar block, lung surgery, postoperative pain

## Abstract

Introduction

A retrolaminar block (RLB) is a modified paravertebral technique with a local anesthetic injected at the retrolaminar site. The aim of this non-inferiority, parallel-group, prospective, and randomized study was to compare the analgesic efficacy of the paravertebral block (PVB) and RLB after lung surgery.

Methods

Eligible subjects were patients aged more than 20 years, with American Society of Anesthesiologists physical status Ⅰ or II, who were scheduled to undergo video-assisted thoracoscopic surgery (VATS) or limited thoracotomy because of lung disease. Patients were randomly allocated to receive either a PVB or RLB using a computer-generated sequence and sealed opaque envelopes. The PVB and RLB were induced by injecting 20 mL of 0.50% ropivacaine and 40 mL 0.25% ropivacaine, respectively. As the primary outcome variable, we considered the area under the curve (AUC) of the postoperative pain intensity using the trapezoidal method. Pain intensity was assessed using an 11-point numerical rating scale (NRS). We converted the NRS (0-10) into the visual analog scale (VAS) (0-100 mm) proportionally. We compared the AUC of the converted NRS (AUC-cNRS) on coughing between one and two hours after the operation. The non-inferiority margin was set at 25 mm × h in the AUC-cNRS. Patients and nurses were blinded to group assignments. Secondary outcomes included time to perform the block, NRS for pain intensity at rest and on coughing at one, two, four, 24, and 48 hours after the operation, the incidence of postoperative nausea and vomiting, time to first morphine use after the operation, and cumulative morphine consumption at 24 and 48 hours after the operation.

Results

In each group, 25 patients were randomized and analyzed. No significant difference in the AUC-cNRS was noted between the groups (P = 0.117). The mean difference in the AUC-cNRS (group RLB minus group PVB) was 13.42 mm × h, 95% confidence interval, −3.48 to 30.32 mm × h. However, when patients with unexpectedly extended skin incision were excluded from the analysis, the AUC-cNRS of group RLB was significantly higher as compared to group PVB (P = 0.0388). The time to perform the block was longer in PVB as compared to the RLB group (P < 0.0001). No significant differences were noted in the remaining secondary outcomes.

Conclusion

The non-inferiority of RLB as compared to PVB was not confirmed. Though RLB has the advantage of a shorter time to perform, RLB is not recommended for patients undergoing VATS or limited thoracotomy because of lack of efficacy as compared to PVB.

## Introduction

For pain relief after surgery, a thoracotomy may necessitate the greatest analgesic strategy among all surgical procedures [1]. Even after thoracoscopic surgery, which is assumed to be less traumatic compared to thoracotomy, postoperative pain can be severe [2]. For pain control after thoracic surgery, whether thoracotomy or thoracoscopy, a single agent or method is not sufficient and multimodal analgesia is a necessity [3-5]. As part of a multimodal analgesic strategy, thoracic epidural analgesia has long been regarded as the gold standard for pain relief after thoracic surgery. However, systematic reviews have demonstrated that thoracic paravertebral analgesia provides comparable outcomes with epidural analgesia after surgery [3,6-8] but has a better side-effect profile, including less postoperative pulmonary complications, nausea, and vomiting [3,8]. Thus, paravertebral analgesia is an attractive alternative to epidural analgesia [3]. However, a paravertebral block (PVB) is accompanied by the risk of a pleural puncture, especially if performed by less skilled doctors.

In 2006, a modified paravertebral technique was proposed as an alternative to the classic PVB approach [9]. In this technique, the needle does not physically enter the paravertebral space and the injectate is placed at the retrolaminar site. Taking this into consideration, Voscopoulos et al. suggest the name retrolaminar block (RLB) for this technique [10]. RLB would logically offer the advantage of a lower risk of pleural injury since needle insertion is made at a more medial puncture site, avoiding needle advancement and manipulation close to the pleura. This approach is proposed as not only safe but also an easy, fast, and effective alternative to other described paravertebral analgesic techniques [9-10].

There are case series or reports that showed the efficacy of RLB in patients undergoing breast surgery [11] or suffering from multiple rib fractures [10]. However, RLB was performed on only one patient undergoing thoracotomy [12]. In general, the internal validity of such case series or reports is very low because of the lack of a comparator group. In addition, selection bias can be problematic. Therefore, the analgesic efficacy of RLB for patients undergoing thoracic surgeries is yet to be determined. The aim of this study was to confirm the non-inferiority of RLB to PVB after less-invasive lung surgery.

This article was previously presented as a meeting poster at the Euroanaesthesia Annual Scientific Meeting on June 4, 2017.

## Materials and methods

Trial design and participants

This was a non-inferiority, parallel-group, randomized, and patient and observer-blinded study with a 1:1 allocation. The study was registered before patient enrolment at www.umin.ac.jp/ctr/index.htm: UMIN000015589. Written informed consent was obtained from all participants. This article adheres to an extension of the CONSORT (Consolidated Standards of Reporting Trials) for reporting noninferiority and equivalence trials (Extension of the CONSORT 2010 Statement).

Eligible subjects were patients aged more than 20 years, with American Society of Anesthesiologists (ASA) physical status Ⅰ or II, who were scheduled to undergo video-assisted thoracoscopic surgery (VATS) or limited thoracotomy because of lung disease at the Hyogo Prefectural Amagasaki General Medical Center. Exclusion criteria included patients who refused to consent, had a known allergy to ropivacaine or flurbiprofen, or had a history of bronchial asthma. At the preoperative visit, each patient was instructed on how to use the patient-controlled analgesia (PCA) device.

Intraoperative management

All patients enrolled in the study received a standardized anesthetic regimen. Standard ASA monitors (electrocardiogram, noninvasive arterial blood pressure, and oxygen saturation) were applied. The application of invasive arterial blood pressure was at the discretion of the anesthesiologist. General anesthesia was induced with intravenous propofol 1.5-2 mg/kg, fentanyl 100 µg, and rocuronium 0.6 mg/kg. Then, a double-lumen endobronchial tube was placed. General anesthesia was maintained with oxygen/desflurane 5%-7%, remifentanil, and rocuronium. After positioning a patient in the lateral position, the surgical procedure was commenced. The same team of surgeons always performed all surgical procedures. In VATS, three thoracoscopy ports were used. In limited thoracotomy, a skin incision (≤5 cm) was made in the midaxillary line. The dermatome of all skin incisions, whether in VATS or in limited thoracotomy, ranged from T4 to T7. During chest closure, chest tube placement was performed with the tube directed posteriorly and inserted up to the apex of the operative side. At the same time, intravenous fentanyl 100 µg and flurbiprofen 50 mg were administered.

Before emergence from anesthesia, patients were randomly allocated to receive either an RLB (group RLB) or a PVB (group PVB) based on a computer-generated sequence and sealed opaque envelopes. To reduce the risk of complications, as well as to ensure better delivery of the local anesthetic, we performed real-time ultrasound-guided blocks. All blocks were performed by an attending anesthesiologist (S.T.) or by anesthesia fellows with more than two years of experience in anesthesia and significant previous experience in ultrasound-guided techniques, under the supervision of the attending anesthesiologist. In both groups, the spinous process of T6 was identified by palpating and counting down from the vertebra prominens (C7) with the patient in the lateral position. After the spinous process of T6 was marked, the skin was disinfected three times. We scanned the paramedian anatomical landmarks of T6, ipsilateral to the surgical side, using an 8-13 MHz linear array ultrasound transducer probe (12-l, Logiq e; GE Healthcare, Tokyo, Japan) placed in a sterile sleeve.

In group RLB, the probe was positioned in the vertical plane approximately 1.5 cm lateral to the spinous process to allow a paramedian sagittal view of the laminae. A 17-gauge, 80 mm, Tuohy needle (Univer; Unisis, Tokyo, Japan) was introduced in an in-plane approach toward the lamina and advanced in a cephalad orientation until contact with the lamina [10] at T6. After a negative aspiration test, the RLB was induced by injecting 40 mL of 0.25% ropivacaine.

In group PVB, the probe was positioned in the vertical plane approximately 2-2.5 cm lateral to the spinous process to allow a paramedian sagittal view of the transverse processes, parietal pleura, and superior costotransverse ligaments. A Tuohy needle was introduced in an in-plane approach toward the paravertebral space [13-14] and advanced in a cephalad orientation to puncture the superior costotransverse ligament at T6. After negative aspiration test results, the PVB was induced by injecting 20 mL of 0.50% ropivacaine.

Average intraoperative remifentanil dosage, duration of operation, and duration of anesthesia were recorded.

Postoperative management

After the operation, all patients stayed in the intensive care unit during the first postoperative night and were returned to the thoracic ward on the first postoperative day. Patients were encouraged to take supplementary doses of morphine from a PCA device (CADD-Legacy; Smiths Medical, Tokyo, Japan). The PCA device was programmed to provide a bolus dose of morphine 1 mg with a 10-minute lockout time, a maximal dose of 4 mg per hour, and no background infusion. As an antiemetic, droperidol 0.05 mg per milligram of morphine was added. In addition, all patients received intravenous flurbiprofen 50 mg at six and 12 hours after the operation and oral loxoprofen 50 mg three times a day on the first and second postoperative days. Intravenous metoclopramide 50 mg was administered on demand for nausea and/or vomiting.

Postoperative pain intensity was evaluated on coughing and at rest at one, two, four, 24, and 48 hours after the operation. Pain intensity was assessed using an 11-point numerical rating scale (NRS; 0 = no pain, 10 = worst pain imaginable) instead of the visual analog scale (VAS) because some patients might be unable to score their pain on the VAS. Postoperative nausea and vomiting were also assessed at the same time points. The postoperative assessments were performed by nurses who were blinded to group allocation. The attending anesthesiologist and anesthesia fellows were not involved in the postoperative patient care and assessment. The hospital stay after surgery and complications such as pneumothorax or local anesthetic toxicity were also recorded.

Primary outcome and sample size

**Figure 1:**
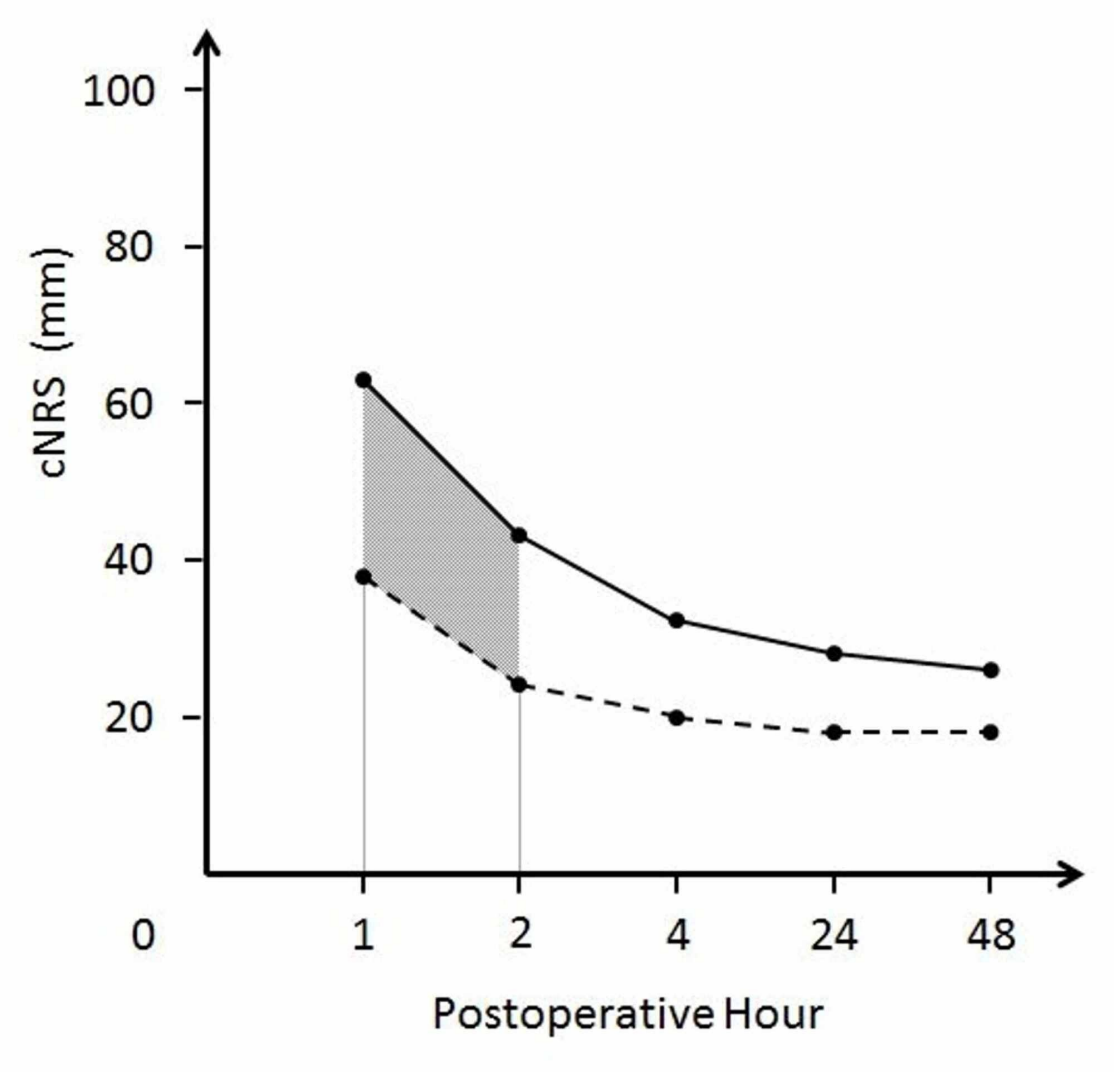
A supplemental diagram of the primary outcome Two lines indicate the imaginary time courses of postoperative pain intensity. The solid line indicates the converted numerical rating scale (cNRS) of the retrolaminar group and the dashed line indicates the cNRS of the paravertebral group. The gray area indicates the difference in the area under the curve of the cNRS between one and two hours.

As the primary outcome variable, we considered the area under the curve (AUC) of the postoperative pain intensity using the trapezoidal method. Since the pain intensity was often measured using the VAS in previous reports, we converted the observed NRS (0-10) into VAS (0-100 mm; 0 mm = no pain, 100 mm = worst pain imaginable) proportionally. Considering that patients suffered from moderate or severe pain immediately after thoracoscopic surgery [2], we compared the AUC of the converted NRS (AUC-cNRS) on coughing between one and two hours after surgery (Figure 1).

Based on previous studies that evaluated pain after thoracoscopic surgery [15-16], the non-inferiority margin was set at 25 mm × h in the AUC-cNRS. Using data from studies by Perttunen et al. [2] and Kaiser et al. [17], which evaluated pain scores after lung surgery, we assumed the AUC-cNRS of group PVB as 50 mm × h. Based on the VAS after thoracotomy [18], the standard deviation of VAS was 20 mm; the calculated standard deviation of AUC-cNRS was 28.3 mm × h.

We calculated that at least 21 patients would be required per group for an experimental design incorporating two groups, with α = 0.05 and β = 0.2 using STATA ver. 13.0 (Stata Corp., College Station, TX). To minimize any effect of data loss, we elected to recruit 50 patients into the study. Non-inferiority was considered if the upper limit of the two-sided 95% confidence interval (CI) for the mean difference in the AUC-cNRS between the groups (group RLB minus group PVB) did not exceed the non-inferiority margin of 25 mm × h.

Secondary outcomes

Secondary outcomes included NRS for pain intensity on coughing and at rest at one, two, four, 24, and 48 hours after the operation and the incidence of postoperative nausea and vomiting. The time required from needle insertion to completion of anesthetic injection was recorded as the time to perform the block. The time to first morphine use after the operation and cumulative morphine consumption at 24 and 48 hours after operation were transcribed from the PCA device memory.

Statistical methods

Statistical analyses were performed using GraphPad Prism version 6.03 for Windows (GraphPad Software, San Diego, CA). The numerical data of the two groups were compared using the independent student's t-test or the Mann-Whitney U test, depending on whether the data were distributed normally or not. The χ2 test or Fisher’s exact test, as appropriate, was used to test differences in proportions in categorical variables. A two-way repeated measure analysis of variance (ANOVA) was used for the analysis of NRS for pain intensity on coughing and at rest at one, two, four, 24, and 48 hours after the operation. Missing values of NRS were compensated by the average value at the corresponding time point of the group since replacing the missing values with the worst value, that is, NRS = 10, or the preceding value of the patient was deemed impractical. A p-value < 0.05 was considered statistically significant.

## Results

**Figure 2:**
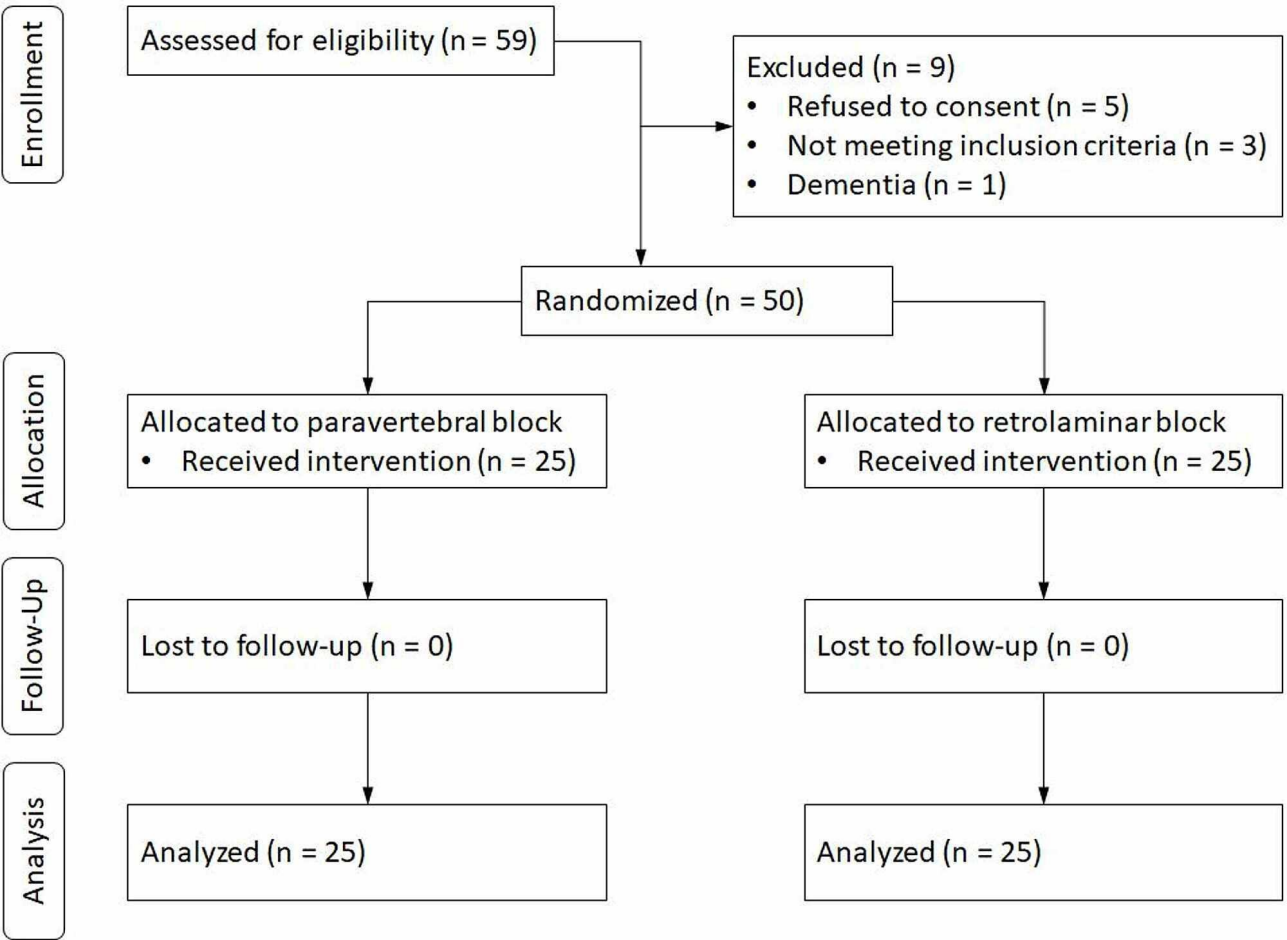
Consolidated Standards of Reporting Trials (CONSORT) statement flow diagram

The CONSORT flow diagram is presented in Figure 2.

Fifty-nine patients were screened for eligibility between November 2014 and July 2016. We excluded nine patients. Of the remaining patients, 25 were randomized to undergo RLB, and 25 were randomized to undergo PVB. The groups were comparable with respect to patient demographics (i.e., age, gender, height, weight, body mass index, and ASA physical status), diagnosis, type of operation, operative procedure, duration of operation and anesthesia, and average remifentanil dosage (Table 1).

**Table 1:** Baseline demographics and surgical characteristics Data are presented as median (interquartile range) or number. ASA: American Society of Anesthesiologists a. The skin incision was unexpectedly extended to 7-8 cm intraoperatively in five patients in group PVB and in two patients in group RLB. The remaining patients underwent limited thoracotomy with skin incision ≤ 5 cm.

	Paravertebral Block	Retrolaminar Block
	(n = 25)	(n = 25)
Age (years)	67 (38–72)	66 (31–73)
Sex		
Male	20	14
Female	5	11
Height (cm)	166 (158–171)	164 (158–167)
Weight (kg)	61 (54–67)	56 (53–62)
Body mass index (kg/m^2^)	22 (21–24)	21 (20–23)
ASA physical status		
I	5	7
II	20	18
Diagnosis		
Pneumothorax	13	10
Cancer	8	14
Benign tumor	4	1
Type of operation		
Thoracoscopy	9	9
Thoracotomy^a^	16	16
Operative procedure		
Bullectomy	10	8
Loop ligation	3	2
Wedge resection	12	14
Segmentectomy	0	1
Duration of operation (min)	103 (83–138)	99 (85–119)
Duration of anesthesia (min)	204 (188–237)	193 (177–209)
Average remifentanil dosage (µg/kg/min)	0.27 (0.22–0.36)	0.24 (0.19–0.32)

The surgery was completed uneventfully. We confirmed the proper placement of the injectate on ultrasound images in all patients. Missing values of NRS were identified in one patient in each group.

Primary outcome

**Figure 3:**
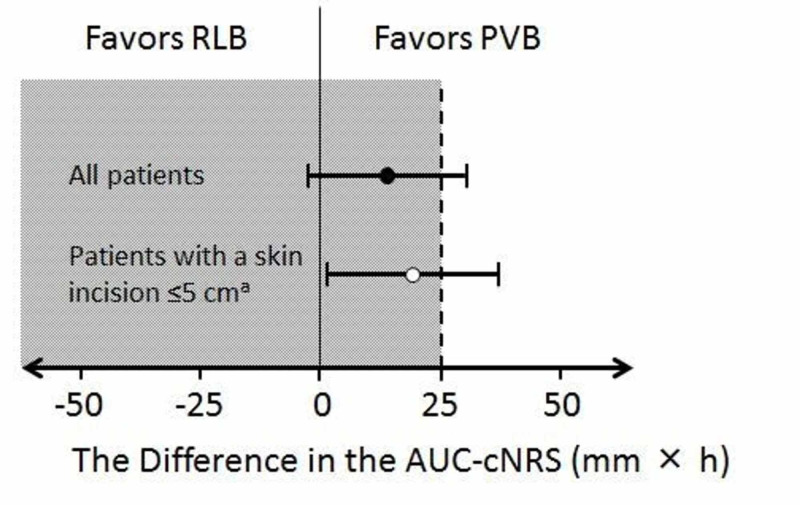
Confidence intervals of the difference in the area under the curve of the converted numerical rating scale (AUC-cNRS) between the groups Error bars indicate two-sided 95% confidence intervals. The dashed line indicates the non-inferiority margin. The gray area to the left of the difference = 25 indicates values for which a retrolaminar block (RLB) would be considered non-inferior to a paravertebral block (PVB). a. Five patients were excluded in group PVB and two in group RLB.

No significant difference in the AUC-cNRS was noted between the groups (group RLB vs. group PVB, mean (SD): 59.00 (27.84) vs. 45.58 (31.48) mm × h, p = 0.117). The mean difference in the AUC-cNRS between the groups was 13.42 mm × h, 95% CI, −3.48 to 30.32 mm × h (Figure 3).

Though thoracotomy with a skin incision >5 cm was not included in our inclusion criteria, the skin incision was unexpectedly extended to 7-8 cm intraoperatively in two patients in group RLB and in five patients in group PVB. When these patients were excluded from the analysis, the AUC-cNRS of group RLB was significantly higher as compared to group PVB [58.04 (27.42) and 39.48 (29.60) mm × h respectively, p = 0.0388]. The mean difference in the AUC-cNRS between the groups was 18.57 mm × h, 95% CI, 1.00-36.14 mm × h (Figure 3).

Secondary outcomes

**Figure 4:**
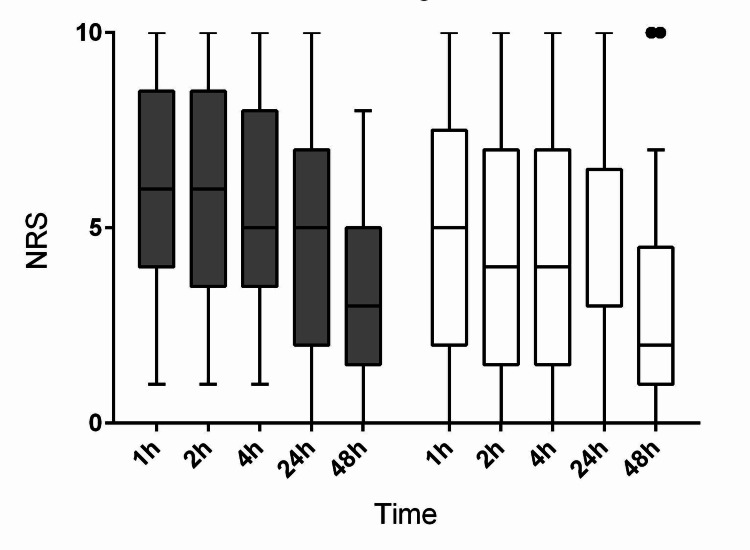
The time course of postoperative pain intensity on coughing Pain intensity was assessed using an 11-point numerical rating scale (NRS). Filled bars indicate the NRS of the retrolaminar group. Open bars indicate the NRS of the paravertebral group.

**Figure 5:**
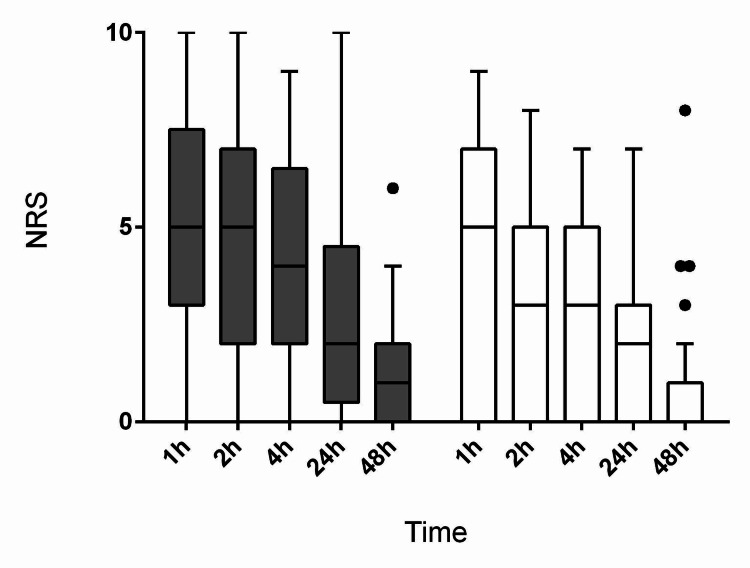
The time course of postoperative pain intensity at rest Pain intensity was assessed using an 11-point numerical rating scale (NRS). Filled bars indicate the NRS of the retrolaminar group. Open bars indicate the NRS of the paravertebral group.

The time course of pain intensity (NRS) after surgery on coughing and at rest is shown in Figure 4 and Figure 5, respectively.

No differences in the intensity of pain measured both on coughing and at rest were reported between the groups (p = 0.1129 and p = 0.2335, respectively; two-way repeated-measures analysis of variance (ANOVA)). The time to perform the block was shorter in group RLB as compared with group PVB (Table 2).

**Table 2:** Postoperative data Data are presented as median (interquartile range) or number. PONV: postoperative nausea and vomiting * Statistically significant difference between groups

	Paravertebral Block	Retrolaminar Block	P Value
	(n = 25)	(n = 25)	
Time to perform block (s)	141 (115–276)	60 (43–87)	<0.0001*
Time to first morphine use (min)	45 (20–144)	22 (5.5–122)	0.26
Morphine use in the first 24 h (mg)	6 (3–14)	9 (4–17)	0.38
Morphine use in the first 48 h (mg)	9 (3–19)	13 (4.5–19.5)	0.68
Incidence of PONV	5	9	0.35
Hospital stay after surgery (d)	4 (4–6)	4 (4–5)	0.96

No significant differences were noted in the remaining secondary outcomes. No severe complications occurred in either group.

## Discussion

When all patients were included in the analysis, the difference in the AUC-cNRS was not significant between the groups. The non-inferiority of RLB as compared to PVB was not confirmed since the upper limit of the 95% CI for the mean difference was 30.32 mm × h in the AUC-cNRS, which exceeded the non-inferiority margin (25 mm × h). On the contrary, when patients with extended skin incision were excluded from the analysis, the AUC-cNRS was significantly higher in group RLB as compared to group PVB. This is probably because the skin incision was extended in more patients in group PVB and they all suffered from severe postoperative pain.

To the best of our knowledge, this is the first randomized trial comparing the analgesic efficacy of RLB and PVB in patients undergoing lung surgery. In this study, we evaluated the analgesic efficacy of a single block rather than a continuous block since a continuous retrolaminar block is not a standardized method. Thus, we limited the subjects to patients undergoing less-invasive lung surgery, such as VATS or limited thoracotomy. In this study, randomization was performed after the end of surgery; this eliminates the possibility that intraoperative anesthetic management affected the results. In addition, nurses who performed postoperative assessments and patients were blinded to group allocation. Thus, the results or differences are assumed to be because of the block technique. In this study, there were several different practitioners who performed the blocks. This means that our results were not owing to a specific practitioner. We consider that having multiple practitioners in our study is favorable with respect to the applicability of our results to other institutions.

Our results indicated that the immediate postoperative analgesic efficacy of PVB was superior as compared to RLB in patients undergoing VATS or limited thoracotomy. One possible explanation for the limited efficacy of RLB is that paravertebral injections made in the dorsal part of the endothoracic fascia resulted in limited longitudinal multisegmental distribution, whereas injections made in the ventral part of the endothoracic fascia resulted in more extensive longitudinal distribution [19]. If this is also the case with RLB where the injectate is placed in a more superficial plane, multiple injections may be required to enhance the segmental extension of the injectate and provide sufficient analgesia after lung surgery. Another explanation could be the lower concentration of ropivacaine (0.25%) used in group RLB as compared to the concentration (0.5-0.75%) in previous reports [10-12]. Juttner et al. used a local anesthetic volume of 40 mL to ensure sufficient craniocaudal spread of anesthesia deriving from a single retrolaminar injection [11]. However, we considered injecting 40 mL of 0.5% ropivacaine could increase the risk of possible local anesthetic toxicity. Therefore, half the concentration and twice the volume of ropivacaine is used in group RLB as compared to group PVB. To date, there are no published data that describe an optimal volume or concentration of local anesthetic for a single injection with RLB. We believe that the lower concentration of ropivacaine in group RLB does not have much impact on the results since patients in both groups received identical dosages of ropivacaine.

In this study, most patients underwent VATS or limited thoracotomy, and these procedures are assumed to be less traumatic than standard thoracotomy. However, patients experienced moderate or even severe pain immediately after surgery. Our results are in line with the findings of a previous study showing that patients experienced moderate or severe pain even after VATS [2]. Increased acute pain may not only impair respiratory function and lead to pulmonary complications such as atelectasis and pneumonia [20] but also increase the incidence of chronic pain [21]. Therefore, adequate analgesia is important even after less traumatic thoracic procedures.

In this study, the time to perform a block was shorter in group RLB as compared to group PVB. This is mainly due to the clear ultrasonographic visualization of the lamina without difficulty and the simple needle advancement until the lamina is reached in group RLB. In contrast, the acoustic windows between transverse processes are narrow, making visualization of paravertebral anatomy and needle advancement elaborate in group PVB.

The mechanism of how the local anesthetic injected at the retrolaminar site exerts analgesic efficacy is inconsistent. Juttner et al. [11] and Pfeiffer et al. [9] hypothesized that the superior costotransverse ligament, which posteriorly delineates the thoracic paravertebral space, is porous enough for the local anesthetic to penetrate into the paravertebral space. On the other hand, Voscopoulos et al. hypothesized that the local anesthetic trickles through the medial aperture of the superior costotransverse ligament or tracks anteriorly through the looser tissues lateral to the facet joint [10]. To date, the actual distribution of the injectate with RLB is not well-studied and further research, including with the use of a contrast agent or dye, is required.

There are several limitations in this study. First, this study has a mixed sample of patients who underwent VATS and limited thoracotomy. Considering that VATS is assumed to be less traumatic and painful as compared to limited thoracotomy, the non-inferiority of RLB might be more likely to be confirmed when two regional techniques are compared only in patients undergoing VATS. However, comparing the efficacy of RLB and PVB in patients undergoing VATS was considered inappropriate since only nine patients in each group underwent VATS. Considering the inherent possibility that VATS unexpectedly converts to limited thoracotomy, comparing the efficacy of two techniques only in patients undergoing VATS would rather impair clinical applicability. Second, since the dermatomal distribution of RLB or PVB was not assessed in this study, we cannot assume the optimal anesthetic volume to cover the skin incisions in less-invasive lung surgery. Also, a third sham group in which we deliver normal saline instead of local anesthetic would have provided additional findings of the analgesic efficacy of RLB. However, we considered that performing a sham block would be inappropriate due to an ethical reason. Third, the generalizability of results deriving from a single-center trial is potentially limited. Though we followed pre-specified perioperative patient care and assessment, our results may not be applicable to other clinical settings.

## Conclusions

Non-inferiority of RLB compared to PVB was not confirmed. RLB has the advantage of a shorter time to perform. However, RLB may not be recommended for patients undergoing VATS or limited thoracotomy because of a lack of efficacy as compared to PVB.
